# Lasting effects of stress physiology on the brain: Cortisol reactivity during preschool predicts hippocampal functional connectivity at school age

**DOI:** 10.1016/j.dcn.2019.100736

**Published:** 2019-11-14

**Authors:** Sarah L. Blankenship, Morgan Botdorf, Tracy Riggins, Lea R. Dougherty

**Affiliations:** a Department of Psychology, University of Maryland, College Park, MD, USA; b Neuroscience and Cognitive Science Program, University of Maryland, College Park, MD, USA

**Keywords:** Stress, Cortisol reactivity, Hippocampus, Passive-viewing fMRI, Functional connectivity, Childhood

## Abstract

Prolonged exposure to glucocorticoid stress hormones, such as cortisol in humans, has been associated with structural and functional changes in the hippocampus. The majority of research demonstrating these associations in humans has been conducted in adult, clinical, or severely maltreated populations, with little research investigating these effects in young or more typically developing populations. The present study sought to address this gap by investigating longitudinal associations between preschool (3−5 years) and concurrent (5–9 years) cortisol reactivity to a laboratory stressor and hippocampal functional connectivity during a passive viewing fMRI scan. Results showed that, after controlling for concurrent cortisol reactivity, greater total cortisol release in response to a stressor during preschool predicted increased anterior and posterior hippocampal connectivity with the precuneus and cingulate gyrus at school-age. These findings are consistent with literature from adult and non-human investigations and suggest lasting impacts of early stress physiology on the brain.

## 1 Introduction

Glucocorticoid stress hormones, such as cortisol, are released as part of a normal physiological response to stress through the functions of the hypothalamic-pituitary-adrenal (HPA) axis. These stress hormones are beneficial to humans when released at optimal times and allowed to fluctuate normally as they enable adaptive coping to stressors (Smith and Vale, 2006). However, with exposure to chronic stress, genetic vulnerabilities (DeRijk, 2009), or disease (Starkman et al., 1992), the HPA axis may become dysregulated, resulting in either heightened or blunted cortisol responses, both in response to a stressor and at baseline (Bunea et al., 2017). Given that dysregulation of the HPA axis is linked to a variety of adverse outcomes, including depression, post-traumatic stress disorder (Jameison and Dinan, 2001), and behavioral deficits (Bolton et al., 2017), it is especially critical to understand the lasting impact of differences in cortisol reactivity during childhood.

Elevated glucocorticoid levels have been associated with changes in both neural structure and function in rodents and nonhuman primates (Sapolsky, 1987; Sapolsky et al., 1990; Uno et al., 1994; for review see Conrad, 2009; Conrad et al., 2017). Impacts of excessive cortisol are particularly evident in regions with high densities of glucocorticoid receptors, such as the hippocampus (Virgin et al., 1991), a structure implicated in an array of processes, including episodic memory, stress regulation, and spatial navigation (Chersi and Burgess, 2015; Herman et al., 2016; Tulving and Markowitsch, 1998). Research using animal models has revealed that exogenous and endogenous exposure to glucocorticoids is linked to structural changes throughout the hippocampus and its subfields (e.g., dentate gyrus; Alfarez et al., 2009; Joëls et al., 1997; Liu et al., 2000). Specifically, excessive levels of cortisol can suppress neurogenesis, inhibit synaptogenesis, and result in atypical dendritic branching and axon development (Gould and Tanapat, 1999; Woolley et al., 1990).

Research examining associations between stress physiology and gross hippocampal structure in humans has yielded findings consistent with those identified in the animal literature at the neuronal level. In particular, associations between cortisol levels and indices of gross hippocampal structure, specifically hippocampal volume, have been investigated. Although the direction of effects is mixed, generally, these studies show associations between cortisol levels and hippocampal volume in human children (Blankenship et al., 2019; Dahmen et al., 2018; Pagliaccio et al., 2014; Wiedenmayer et al., 2006), young adults (Narita et al., 2012; Pruessner et al., 2007), older adults (e.g., Sudheimer et al., 2014), and individuals with stress-related disorders (e.g, Nelson and Tumpap, 2017).

Importantly, cortisol-related structural changes in the hippocampus have functional implications. For example, rodents exposed to elevated levels of glucocorticoids not only show differences in neural structure, but also exhibit differences in neuronal excitability in the hippocampus (Joels and de Kloet, 1989; Okuhara and Beck, 1998). In humans, cortisol reactivity likely impacts hippocampal function, as well; however, only limited research has examined functional changes in the hippocampus related to cortisol reactivity. Most of this research has focused on adolescent and adult samples, and examined concurrent associations between stress physiology and hippocampal functional connectivity during resting-state functional MRI (e.g., Kiem et al., 2013; Sripada et al., 2014). One study assessed hippocampal functional connectivity in a sample of young adolescents (7−15 year-olds, *M* = 11.8 years; Thomason et al., 2013) and found greater concurrent cortisol reactivity was associated with greater hippocampal connectivity with the default mode network (DMN), a group of neural regions active in the absence of a task (Greicius et al., 2003). However, other studies assessing cortisol reactivity in adult populations have failed to find similar relations (e.g., Kiem et al., 2013; Sripada et al., 2014). For example, in a sample of adults, Sripada et al. (2014) found that greater concurrent cortisol reactivity did not predict hippocampal connectivity with any region, but lower childhood income (a proxy for childhood stress) predicted reduced hippocampal connectivity with regions in the DMN. Together, these results suggest age-related differences in the effect of stress reactivity on hippocampal functional connectivity, which may ultimately be important to behavioral outcomes or the emergence of stress-related disorders later in development.

Although there is limited research in adolescents and adults, no studies have assessed cortisol reactivity and hippocampal functional connectivity in young children or the potential age-dependence of this association in early (e.g., before 5 years) versus later (e.g., after 5 years) childhood. This is a significant gap in the literature, as impacts of cortisol reactivity on hippocampal connectivity may be stronger during early childhood when the hippocampus is undergoing rapid development and is particularly susceptible to environmental influences (Andersen et al., 2008; Gogtay et al., 2006; Humphreys et al., 2019; Tottenham & Sheridan 2009). In addition, the hippocampus is part of a wider network of brain regions (Riggins et al., 2016; Vincent et al., 2006), and research shows that a great deal of refinement of network connectivity occurs in early childhood (Johnson, 2001). Network development is impacted by environmental factors, such as psychosocial stress (Lipina and Posner, 2012; Teicher, Samson, Andersen, & Ohasi, 2016), which may have important implications for the impact of cortisol on the development of the hippocampus and its connectivity to cortical regions.

Investigating the potential timing-dependent effects of cortisol reactivity on hippocampal functional connectivity is necessary to understand interactions between stress physiology, hippocampal network development, and later risk for stress-related disorders. The current study sought to address critical gaps in the literature by investigating associations between early (3−5 years) and concurrent (5–9 years) cortisol reactivity and hippocampal functional connectivity during a passive viewing paradigm in school-aged children. Based on previous research in adolescents and young adults, we hypothesized that greater cortisol reactivity would be associated with hippocampal connectivity with regions within the DMN. We also hypothesized that early cortisol reactivity would exert a *greater* effect on hippocampal functional connectivity than concurrent cortisol reactivity, given research highlighting the effects of early life stress and stress physiology on the hippocampus.

## 2 Methods

### 2.1 Participants

The current sample was part of a larger longitudinal study (*N* = 175) examining neuroendocrine risk markers for depression in early childhood (for a complete description of study recruitment and eligibility, see Dougherty et al., 2013; Leppert et al., 2016). Children were oversampled for a parental lifetime history of depressive disorders, assessed with the Structured Clinical Interview for DSM-IV Disorders (SCID; First et al., 1996). Children were assessed during preschool (Time 1) and approximately 3 years later (Time 2) to capture the transition to school entry. Every participant who completed a session was invited to attend the subsequent session. At each time point, children and their families completed multiple assessments.

From the full longitudinal sample, 156 children completed the Time 1 cortisol assessment, 104 children completed the Time 2 cortisol assessment, and 64 children attended the Time 2 neuroimaging session. Of the 64 children who attended the neuroimaging session, 42 children provided usable neuroimaging data (1 child attended the neuroimaging session, but did not complete the scan due to claustrophobia; 2 children completed the functional scan with different scan parameters; 4 children did not complete a full functional scan; and 15 children were excluded due to excessive motion during the functional scan, see 2.3 *Neuroimaging Assessment* for discussion of motion considerations). Of these 42 children, 41 also provided usable cortisol reactivity data. Final sample sizes ranged from 38 to 41 subjects for analyses involving both neuroimaging and cortisol reactivity data. Specifically, 39 children provided usable neuroimaging and cortisol reactivity data at Time 1; 40 children provided usable neuroimaging and cortisol reactivity data at Time 2; and 38 children provided usable neuroimaging and cortisol reactivity data at both Time 1 and Time 2.

Table 1 summarizes demographic data for the 41 children included in the present report.

**Table 1:** Demographic characteristics of sample.

Demographic variable	
Child age (in years) at T1 Cortisol Assessment, [Mean (SD), range] (*n* = 39)	4.32 (.81), 3.12-5.97
Child age (in years) at T2 Cortisol Assessment, [Mean (SD), range] (*n =* 40)	7.21 (.74), 5.64-8.70
Child age (in years) at T2 Scan, [Mean (SD), range]	7.51 (.74), 5.93-8.91
Delay (in years) between T1 and T2 Cortisol Assessment, [Mean (SD), range] (*n* = 38)	2.99 (.45), 2.11-3.98
Delay (in days) between T2 Cortisol Assessment and T2 Scan, [Mean (SD), range] (*n =* 40)	103.08 (101.02), 15.99-437.70
Child sex, [*n* (%)]	
Female	22 (53.7%)
Child race, [*n* (%)]	
White, European-American	19 (46.3%)
African American	15 (36.6%)
Asian	0 (0%)
Multi-Racial/Other	7 (17.1%)
Child ethnicity (*n* = 47) [*n* (%)]	
Hispanic/Latino descent	7 (17.1%)
Single parent household [*n* (%)]	
Lives with only one parental figure	7 (17.1%)
Family income [*n* (%)]	
<$20,000	3 (7.3%)
$20,001 to $40,000	2 (4.9%)
$40,001 to $70,000	13 (31.7%)
$70,001 to $100,000	10 (24.4%)
>$100,000	12 (29.3%)
Parental education [*n* (%)]	
At least one parent with a four-year college degree	31 (75.6%)
Maternal lifetime history of depressive disorder [*n* (%)]	
Lifetime history present	21 (51.2%)

*Note*. *n* = 41 unless otherwise noted; T1=Time 1; T2 = Time 2.

This subsample of children did not significantly differ from the full sample on child sex (*χ^2^* (1, *N =* 175) = .186, *p =* .667), family income (*χ^2^* (4, *N =* 167*) =* 4.24, *p =* .374), race (*χ^2^* (4, *N =* 171) *=* 7.80, *p =* .099), ethnicity (*χ^2^* (1, *N =* 169*)* = .017, *p =* .896), parental education (*χ^2^* (1, *N =* 172) *=* .935, *p =* .333), maternal lifetime history of depressive disorder (*χ^2^* (1, *N =* 167*) =*.001, *p =* .971), child age at Time 1, (*t* (171) = -.67, *p* =  .503), or child age at Time 2 (*t* (112) = 0.39, *p* =  .697).

### 2.2 Cortisol reactivity assessments

Age-appropriate stressor tasks were administered at both Time 1 and Time 2. During both assessments, the experimenter pretended to take notes on the child’s performance to elicit feelings of social evaluation. At Time 1, an existing laboratory stress paradigm that has been successful in eliciting a cortisol response in preschool-aged children was used (Kryski et al., 2011; Lewis and Ramsay, 2002). The task is a developmentally-appropriate laboratory stressor, which requires children to pair animal pictures with colored chips within 3 min. Children were told that the task was easy for young children and that they would receive a desired prize based on their performance. The experimenter manipulated a timer so the child failed to complete the task three times. After the third failed trial, the experimenter informed the child that the timer was broken, praised the child’s performance, and presented the child with a desired prize (for additional task details, see Dougherty et al., 2013; Tolep and Dougherty, 2014).

At Time 2, children completed a modified version of the Trier Social Stress Task for Children (TSST-C; Buske-Kirschbaum et al., 1997) followed by an unsolvable puzzle. Children selected a favorite and a least favorite prize and were told that a judge would evaluate their performance on the tasks to determine which prize the child received. First, children were instructed to tell a 4.5-minute story about an unfamiliar picture book after 30 s of preparation. After the story telling task, children were instructed to complete an unsolvable puzzle that contained pieces from two highly similar, but different, puzzles within 3 min (for a complete description of the tasks, see Leppert et al., 2016). After the task, the experimenter explained that the child performed well, informed the child of the mixed-up puzzle pieces, apologized for the error, and presented the child with the desired prize.

At both Time 1 and Time 2, children provided five salivary cortisol samples, including a baseline sample collected before the stressor after 30 min of quiet play and four samples after the stressful task (at 20-, 30-, 40-, and 50-min post-stressor). To collect salivary cortisol samples, a cotton roll was dipped in a small amount of Kool Aid (.025 mg) and placed in the child’s mouth until saturated with saliva (∼1 min). This method, which does not influence cortisol assays if used sparingly and consistently, stimulates saliva production and makes the sampling procedure more pleasant for young children (Talge et al., 2005). Cortisol samples were frozen at −20 °C, and assayed in duplicate using a time-resolved fluorescence immunoassay with fluorometric end-point detection (DELFIA) at the Biochemical Laboratory at the University of Trier, Germany. Inter- and intra- assay coefficients of variation ranged between 7.1–9.0% and 4.0–6.7%, respectively.

Cortisol reactivity in response to the laboratory stressor was captured using two indices: area under the curve with respect to ground (AUCg) and area under the curve with respect to increase (AUCi). AUCg and AUCi measure distinct aspects of the cortisol response to a stressor. AUCg provides a measure of the total amount of cortisol released in response to a stressor, while AUCi provides a measure of total cortisol change from each subject’s individual baseline in response to a stressor. These measures were derived from each participant’s 5 cortisol samples using formulas detailed in Pruessner et al. (2003).

One participant had at least two extreme cortisol samples at Time 2 (exceeded 44 nmol/L) and was thus excluded from analyses. Final AUCg and AUCi values were log-transformed and z-scored. Time 1 and Time 2 AUCg and AUCi were not correlated (*r*_AUCg_ = .21, *p* =  .20; *r*_AUCi_ = .15, *p* =  .36), consistent with previous reports utilizing the same sample, which indicated lack of stability of the cortisol response over time (Leppert et al., 2016). As reported in Leppert et al. (2016), 47 % of children were considered cortisol “responders” (i.e., those whose cortisol levels increased by over 10 % from baseline to the peak post-stressor value) to the laboratory stressor at Time 1, and 60.8 % of children were considered cortisol “responders” at Time 2.

### 2.3 Neuroimaging assessment

At the Time 2 neuroimaging assessment, children first completed training in a mock scanner to become acclimated to the scanner environment and receive feedback regarding motion. Participants were then scanned in a Siemens 3.0-T scanner (MAGNETOM Trio Tim System, Siemens Medical Solutions, Erlangen, Germany) using a 12-channel coil. Children watched a video of their choosing while structural data were collected using a high-resolution T1 magnetization-prepared rapid gradient-echo (MPRAGE) sequence consisting of 176 contiguous sagittal slices (1.0 × 1.0 × 1.0 mm voxel dimensions; 1900 ms TR; 2.52 ms TE; 900 ms inversion time; 9° flip angle; pixel matrix = 256 × 256). Next, children completed a functional passive viewing scan, which operates under the same assumptions of a resting state scan, but instead of viewing a fixation cross, participants passively view a movie or screensaver (Vanderwal et al., 2015). Importantly, metrics of functional connectivity during passive viewing conditions can be used to predict meaningful individual differences in cognitive and behavioral performance (Riggins et al., 2016). During the functional scan, children passively viewed abstract shapes (similar to screen savers) for 6 min. Data were collected with the following scan parameters: 180 EPI volumes consisting of 36 oblique interleaved slices with a 3.0 × 3.0 × 3.0 mm voxel size; 2 s TR; 24 ms TE; 3 mm slice thickness; 90° flip angle; 64 × 64 pixel matrix.

### 2.4 Neuroimaging data processing

T1 images were analyzed in Freesurfer (Version 5.1.0), an automatized segmentation package (surfer.nmr.mgh.harvard.edu). Freesurfer segmentation files were used to generate subject-specific masks for hippocampal and nuisance signal timeseries extraction. Hippocampal segmentations were visually checked and manual edits were performed (*n* = 5), as necessary, to correct for gross over- or under-inclusions. Hippocampal masks were segmented into anterior and posterior segments given evidence of differential functional connectivity between these subregions (Blankenship et al., 2017; Poppenk et al., 2013). This was achieved by first aligning Freesurfer volumes to the anterior commissure-posterior commissure to eliminate distortions introduced by reorientation (Poppenk and Moscovitch, 2011). The posterior boundary of the anterior hippocampus was identified as the last coronal slice in which the uncal apex was visible (Riggins et al., 2015; Weiss et al., 2005). Two independent raters identified this division for each scanned participant with a high degree of consistency between raters. Intraclass correlation coefficients (ICCs) for the posterior boundary of the right and left anterior hippocampus were .92 and .94, respectively.

Resulting left and right hippocampal masks were combined into a single bilateral anterior and a single bilateral posterior seed for each individual. Bilateral seeds are a common methodological choice to limit the number of analyses run in under-powered samples. Additional masks were generated from the Freesurfer subcortical segmentation for left and right hemisphere white matter, corpus callosum, and lateral ventricles. Each mask was resampled to functional resolution and clipped at 100 %, 50 %, 80 %, and 90 %, respectively, for cerebral white matter, corpus callosum, lateral ventricles, and all hippocampal seeds.

Functional images were slice-time corrected in the Analysis of Functional NeuroImages software package (AFNI; Version 16.0.00; Cox, 1996). All functional images were aligned to the first volume using rigid-body motion-correction. Functional data was then registered to both the T1 structural image and the Freesurfer subcortical segmentations using the Advanced Normalization Tools software (ANTs; version 1.9.v4; http://stnava.github.io/ANTs/). The data were then bandpass filtered at .009 < *f* < .08 and nuisance regressed. Nuisance regression included 21 regressors: 6 motion parameters and their 6 temporal derivatives; baseline, linear, quadratic, and cubic drift; and separate timeseries for left and right hemisphere white matter, left and right hemisphere lateral ventricles, and the corpus callosum. One participant’s lateral ventricles were too small to generate lateral ventricle masks that did not intersect with surrounding neural tissue. Therefore, nuisance regression for this participant did not include CSF regressors.

Functional volumes were then normalized to a 4.5–8.5 year symmetrical MNI Child Template (Fonov et al., 2011) with a multivariate transformation in ANTs. Data were smoothed using a 6 mm Gaussian kernel within a whole brain mask. Whole brain connectivity analyses were run for hippocampal anterior and posterior seeds, separately, using the 3dDeconvolve command in AFNI. The resulting R^2^ values were converted to Pearson’s r and then to z-scores using a Fisher’s r-to-z transformation. Individual subjects’ z-scored connectivity maps were entered into the group analysis. To control for multiple comparisons, 10,000 Monte Carlo simulations were run on the residual timeseries of each analysis using AFNI’s 3dClustSim (Version 17.2.10) for analysis-specific cluster-corrected *p*-values. The spatial autocorrelation in the current sample was first specified using 3dFWHMx. Minimum cluster sizes (k) were calculated for each analysis using the spatial autocorrelation data and ranged in size from 107 to 113 voxels for *p*_corrected_ <.05 at *p*_uncorrected_ <.005.

Motion has been shown to have significant deleterious effects on resting-state analyses, especially in young children who may be susceptible to more frequent and larger movements than adults (Power et al., 2013, 2014; Power et al., 2015; Satterthwaite et al., 2012; Van Dijk et al., 2012). To mitigate any possible effects of motion on our results, a number of precautions were taken: (1) Only participants who showed < 3.0 mm of movement from a reference volume throughout the entire scan were included; (2) volumes demonstrating > 0.5 mm of framewise displacement (FD), calculated as the Euclidean distance from the previous volume, were censored along with the preceding volume; (3) only subjects with at least 4 min of data were included in analyses (Geng et al., 2018); (4) mean FD was calculated for each individual and included in all analyses as a covariate; and (5) we ensured that mean FD did not correlate with age (*r*=-.18, *p* = .272).

### 2.5 Data analytic methods

Analyses were focused on examining timing-dependent differences in the associations between early and concurrent cortisol reactivity (assessed using AUCg and AUCi) with anterior and posterior hippocampal functional connectivity. Main effects of early and concurrent cortisol reactivity were tested first in separate analyses, followed by timing-dependent analyses that controlled for cortisol reactivity at the other time point. AUCg and AUCi were tested in separate analyses.

Mean FD and child age at time of scan were entered as covariates in all analyses to mitigate the effects of individual differences in motion on connectivity metrics and to control for known differences in network connectivity across age. Sex, maternal depression, and parental education were investigated as additional potential covariates. To conserve power in our relatively small sample, whole-brain associations with potential covariates were assessed and only variables that were significantly correlated with the dependent variables (anterior and posterior hippocampal connectivity metrics) were retained as covariates. Specifically, these were variables where clusters were present at *p_voxel-level_* < .05. Resulting covariates included maternal depression, mean FD, and scan age. These covariates were used in all analyses.

Separate regressions were run to test the main effects of Time 1 and Time 2 cortisol reactivity on Time 2 hippocampal connectivity. For each analysis, each individual subject’s z-scored whole-brain anterior or posterior hippocampal functional connectivity maps were entered into AFNI’s 3dttest++ along with Time 1 or Time 2 cortisol reactivity as the predictor and mean FD, scan age, and maternal depression as covariates.

Timing-dependent analyses included both Time 1 and Time 2 AUCg or AUCi as independent variables, which enabled investigation of unique effects of developmental timing. Specifically, these analyses provided the ability to assess whether early or concurrent cortisol reactivity is a stronger predictor of hippocampal functional connectivity. Individual subjects’ z-scored whole-brain anterior or posterior hippocampal functional connectivity maps were entered into AFNI’s 3dttest++ along with Time 1 and Time 2 cortisol reactivity as the predictors and mean FD, age, and maternal depression as covariates.

## 3 Results

**Fig. 1:**
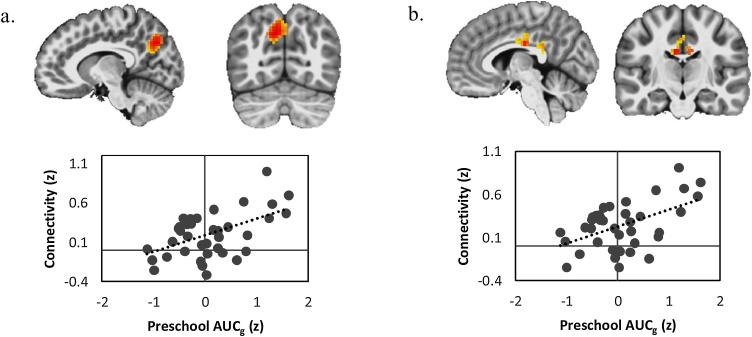
Greater Time 1 total cortisol release (AUCg) in response to a laboratory stressor was significantly associated with greater anterior hippocampus connectivity with, a.) right precuneus/cuneus and b.) right midcingulate cortex (*n* = 39).

Greater Time 1 AUCg significantly predicted increased bilateral anterior hippocampus connectivity with right precuneus/cuneus and right midcingulate cortex (MCC) controlling for scan age, mean FD, and maternal depression (Table 2, Fig. 1). Time 1 AUCg was not a significant predictor of posterior hippocampal connectivity with any region. Neither Time 2 AUCg nor Time 1 or Time 2 AUCi significantly predicted Time 2 anterior or posterior functional connectivity with any neural regions.

**Table 2:** Regions in which bilateral anterior hippocampal functional connectivity significantly varied as a function of Time 1 total cortisol release (AUCg) in response to a laboratory stressor (n=39).

Region	k	x	y	z	t
Right Precuneus	162	11	−71	36	5.00
Right Cuneus					
Right Midcingulate Cortex	144	5	−17	30	4.58

**Fig. 2:**
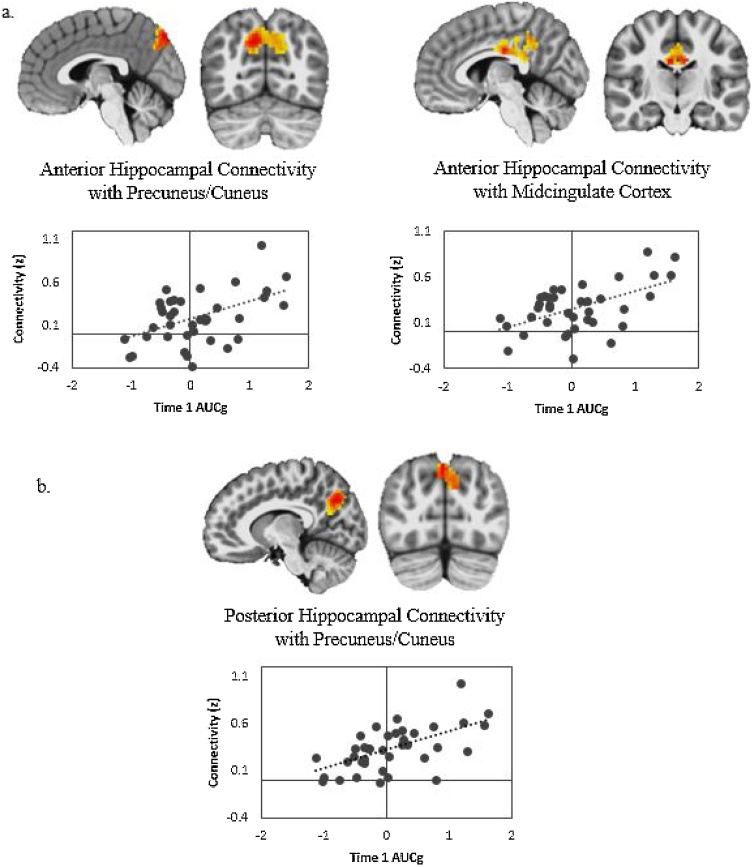
Regions where greater Time 1 total cortisol release (AUCg) in response to a stressor was significantly associated with greater (a) anterior and (b) posterior hippocampus connectivity after controlling for Time 1 cortisol release, mean FD, scan age, and maternal depression (*n* = 38). *Note:* Scatterplots depict bivariate correlations between the predictor and connectivity and are not adjusted for covariates included in the statistical models.

Regressions were run to examine timing-dependent differences in associations between Time 1 and Time 2 AUCg or AUCi and either anterior or posterior hippocampal functional connectivity. Similar to the main effects analyses, greater Time 1 AUCg predicted increased anterior hippocampal connectivity with right precuneus/cuneus and right MCC when controlling for Time 2 AUCg, scan age, mean FD, and maternal depression (Fig. 2a, Table 3). Notably, the timing-dependent analyses also revealed new effects that were not observed in the main effects analyses. Specifically, results showed that greater Time 1 AUCg, controlling for Time 2 AUCg and additional covariates, was related to increased posterior hippocampus connectivity with right precuneus (Fig. 2b, Table 3). There were no significant associations between Time 2 AUCg and bilateral anterior or posterior hippocampal connectivity when controlling for Time 1 AUCg. Furthermore, there were no significant timing-dependent effects of Time 1 or Time 2 AUCi on anterior or posterior hippocampal connectivity with any region.

**Table 3:** Regions in which bilateral hippocampal connectivity varied as a function of Time 1 total cortisol release (AUCg) in response to a stressor, controlling for Time 2 AUCg (n=38).

Region	k	x	y	z	*t*
*Anterior Hippocampus*					
Right Precuneus	312	11	−71	36	5.37
Right Cuneus					
Right Midcingulate Cortex	293	5	−17	30	4.97
*Posterior Hippocampus*					
Right Precuneus	225	2	−80	45	4.99
Left Precuneus					

## 4 Discussion

This is the first study to investigate longitudinal associations between preschool and concurrent cortisol reactivity on hippocampal functional connectivity in a young child population. Analyses revealed associations between total cortisol release in response to a stressor (AUCg) and hippocampal functional connectivity assessed approximately 3 years later. Specifically, results showed that greater preschool cortisol release was related to greater anterior hippocampal connectivity with precuneus and MCC at school-age. Concurrent cortisol release did not significantly predict anterior or posterior hippocampal connectivity with any region of the brain. Results also revealed timing-dependent effects of early cortisol release, controlling for concurrent cortisol release, on anterior and posterior hippocampal connectivity with precuneus and anterior hippocampal connectivity with MCC. Cortisol change in response to the laboratory stressors (AUCi) did not predict hippocampal connectivity, suggesting that greater total cortisol secretion may be more impactful on hippocampal network connectivity than change in cortisol reactivity, especially earlier in development.

In the present study, greater cortisol release was associated with increased hippocampal connectivity with precuneus and MCC, which may indicate that enhanced cortisol secretion early in life is a factor that influences hippocampal connectivity. Research shows that the hippocampus is functionally connected to the precuneus and the cingulate cortex in children (Riggins et al., 2016) and young adults (Poppenk et al., 2013) and that this connectivity increases in magnitude during childhood (Blankenship et al., 2017). These changes in connectivity occur during development as specific networks become more specialized (Johnson, 2001). Importantly, factors such as psychosocial stress, can impact network development as well (Lipina and Posner, 2012; Teicher et al., 2016). Consistent with related research, the current findings suggest that hippocampal network development, in particular, may be accelerated in children who exhibit greater cortisol release (Callaghan and Tottenham, 2016).

Observed cortisol-related differences in hippocampal connectivity likely have implications for cognitive processing. Research shows that functional connectivity between the hippocampus and precuneus and the hippocampus and cingulate cortex relates to memory performance in children (Riggins et al., 2016) and adults (Ranganath et al., 2005; Vincent et al., 2006). Thus, increased connectivity related to heightened cortisol release in childhood may relate to variations in memory ability later in development. Furthermore, precuneus and cingulate cortex are regions within the DMN (Fransson and Marrelec, 2008), and altered activity of this network has been implicated in psychopathology, including depression and post-traumatic stress disorder (Akiki et al., 2018; Sambataro et al., 2014; Whitfield-Gabrieli and Ford, 2012). Therefore, these differences in functional connectivity may have important implications for both cognitive processes, such as memory, and psychopathology. Future research should work to explore these implications.

Notably, in the present study, the effect of cortisol on functional connectivity appears to be specifically driven by cortisol release in early childhood in contrast to release later in middle childhood, suggesting a potential sensitive period for the development of these functional associations. Furthermore, early total cortisol release was related to posterior hippocampal connectivity with precuneus only when taking into account concurrent cortisol secretion, highlighting the importance of investigating unique developmental timing effects. These effects correspond to previous studies suggesting that birth to 5 years old may represent a sensitive period for the effect of stress on the hippocampus (Andersen et al., 2008; Humphreys et al., 2019; Tottenham and Sheridan 2009). However, this is in contrast to the study by Thomason and colleagues (2013), which found a positive association between concurrent cortisol reactivity and hippocampal-DMN connectivity in a slightly older sample of 7–13 year-olds (*M =* 11.1 years). Taken together, these findings suggest that cortisol is likely associated with hippocampal connectivity with precuneus and MCC; however, the timing-dependence of these associations requires further investigation.

In the current study, there was some specificity of results with regard to subregion of the hippocampus. Early cortisol release was related to anterior hippocampal connectivity with MCC, whereas no results were found with posterior hippocampus-MCC connectivity. However, the pattern of effects between early cortisol secretion and hippocampal connectivity with the precuneus was similar for both anterior and posterior hippocampus. These results are consistent with research indicating both distinct and overlapping connectivity between anterior and posterior hippocampus and various regions in the brain (Poppenk et al., 2013). These results also highlight the importance of examining hippocampal subregions rather than examining connectivity with the hippocampus as a whole.

The present study expands upon and makes valuable contributions to the existing literature. Importantly, by investigating hippocampal connectivity, we are able to learn more about changes that extend beyond a particular structure to begin to understand how cortisol impacts the hippocampus on a network level. Second, use of a young, longitudinal sample afforded the capability to probe timing-dependent effects of early and concurrent cortisol reactivity on hippocampal functional connectivity. Third, use of regionally-specific hippocampal seeds permitted examination of distinctions in connectivity between hippocampal subregions. Fourth, this study used strict motion criteria, which increases confidence in the effects presented. Finally, the study used multiple indices of cortisol reactivity and developmentally appropriate stressor paradigms across two developmental periods.

The study also had several limitations. First, the present investigation may not have had sufficient power to detect additional effects. Despite 104 children participating in the Time 2 cortisol assessments, MR contraindications and participant interest in participating in the MRI session significantly reduced the number of participants included in the present analyses. Second, although longitudinal measures of cortisol reactivity were collected, the present investigation only acquired neuroimaging data at Time 2. A single time point of neuroimaging data does not allow for the determination of the temporal relations between variables. It is unknown whether baseline differences in connectivity were present earlier in life or at what point in development individual differences emerged. Additionally, because imaging data was only collected at Time 2, it is not possible to know how long observed effects last or how they may continue to change throughout development. Future investigations should strive to use larger longitudinal samples with more frequent imaging measurements to draw conclusions regarding developmental change and long-term outcomes, including the detection of possible sensitive periods. Finally, given our small sample size, we were unable to assess additional variables, such as sex, that may be important to the association between cortisol and connectivity. Future studies should include interactions with sex, in particular, as research shows that males and females’ hippocampi may be impacted by glucocorticoids in different ways (Liu et al., 2006).

## 5 Conclusion

In sum, the present study found evidence that the development of hippocampal functional connectivity may be shaped by the early cortisol response to stress. Specifically, results demonstrated that greater preschool cortisol secretion predicted greater hippocampal connectivity with distinct neural regions, including precuneus and MCC. Future research should explore immediate and long-term behavioral and cognitive implications of the present findings of early cortisol-related differences in hippocampal connectivity. A complete understanding of these implications will be necessary to determine how reciprocal and timing-dependent associations between stress physiology and patterns of functional connectivity may predict individual differences in cognition or behavior and possible risk for later psychopathology.

## Funding

This research was supported by the Maryland Neuroimaging Center Seed Grant Program (LRD), National Science Foundation in partnership with the University of Maryland ADVANCE Program for Inclusive Excellence (LRD & TR), University of Maryland College of Behavioral and Social Sciences Dean’s MRI Research Initiative RFP Program (LRD & TR), Behavioral and Social Sciences Dean’s Research Initiative (LRD), the Research and Scholarship Award (LRD), University of Maryland Graduate Dean's Dissertation Fellowship (SLB), and National Science Foundation Graduate Research Fellowship Program grant (MB).

## Declaration of Conflict of Interest

The authors report no conflicts of interest.
